# New Region-Scalable Discriminant and Fitting Energy Functional for Driving Geometric Active Contours in Medical Image Segmentation

**DOI:** 10.1155/2014/357684

**Published:** 2014-07-07

**Authors:** Xuchu Wang, Yanmin Niu, Liwen Tan, Shao-Xiang Zhang

**Affiliations:** ^1^Key Laboratory of Optoelectronic Technology and Systems of Ministry of Education, College of Optoelectronic Engineering, Chongqing University, Chongqing 400044, China; ^2^College of Computer and Information Science, Chongqing Normal University, Chongqing 400050, China; ^3^Department of Anatomy, Third Military Medical University, Chongqing 400038, China

## Abstract

We propose a novel region-based geometric active contour model that uses region-scalable discriminant and fitting energy functional for handling the intensity inhomogeneity and weak boundary problems in medical image segmentation. The region-scalable discriminant and fitting energy functional is defined to capture the image intensity characteristics in local and global regions for driving the evolution of active contour. The discriminant term in the model aims at separating background and foreground in scalable regions while the fitting term tends to fit the intensity in these regions. This model is then transformed into a variational level set formulation with a level set regularization term for accurate computation. The new model utilizes intensity information in the local and global regions as much as possible; so it not only handles better intensity inhomogeneity, but also allows more robustness to noise and more flexible initialization in comparison to the original global region and regional-scalable based models. Experimental results for synthetic and real medical image segmentation show the advantages of the proposed method in terms of accuracy and robustness.

## 1. Introduction

Image segmentation is a fundamental topic in image processing and computer vision, object tracking, and medical imaging. The main objective is to partition image signal into different objects thereby separate the components of interest from the background. In the field of medical imaging based diagnosis, image segmentation is very challenging because medical images usually are characterized as nonuniformity of regional intensities, highly complex constructions of tissues and organs, and varying noise. To address them, active contours-based methods have received increasing attention with considerable success [1, 2]. In this study, we focus on geometric active contour models in a level set formulation [3–5].

Geometric active contour model has been proved to be effective for medical image segmentation because it combines the merits of energy functional-driven curve evolution and level set framework. The basic idea is that an active contour is implicitly represented as the zero level set of a function in higher dimension (called level set function, LSF), and then the LSF is deformed according to an evolving partial differential equation (PDE) or variational derived from the energy functional. This implicit model usually outperforms traditional parametric active contour models in that the level set evolution allows for automatic topological change of active contour, such as the contours' merging and splitting during evolution [6].

Existing geometric active contour models can be roughly categorized into two classes: edge-based models [3, 7, 8] and region-based models [9–21] according to image features used for segmentation. Generally, edge-based models typically use image gradient as an image-based force to attract the contour toward object boundaries. These models have been successfully used for general images with strong object boundaries, but they may suffer from boundary leakage problem for objects with weak boundaries, for example, vessel or brain MR images. In contrast, region-based models have better performance in the presence of weak boundaries since they use region's statistics rather than gradient on edges to find a partition of image domain. However, region-based models basically tend to rely on intensity homogeneity. The well-known CV model [9] is based on the assumption that image intensities are statistically homogeneous in each region and therefore fail to segment those images with intensity inhomogeneity. Some improved CV models [10] overcome this limitation by using piecewise smooth description of the images and thus exhibit certain capability of handling intensity inhomogeneity. However, computational cost of the piecewise smooth description is rather expensive due to the complicated procedures involved. This limitation, along with their somewhat complex parameter settings, has made the PS models barely useful for some medical image segmentation.

In fact, intensity inhomogeneity occurs in many real-world images from different modalities [22]. In particular, it is often seen in medical images, such as X-ray radiography/tomography and magnetic resonance (MR) images. For example, the intensity inhomogeneity in MR images often appears as an intensity variation across the image, which arises from radio-frequency coils or acquisition sequences [23]. Thus the resultant intensities of the same tissue vary with the locations in the image. Similar artifacts also occur in CT images due to the beam hardening effect, as well as in ultrasound images caused by nonuniform beam attenuation within the body.

Recently, Li et al. proposed a region-scalable fitting (RSF) model [12, 13] to handle the difficulty in segmentation caused by intensity inhomogeneity. The RSF model constrains the statistics in a local region and draws upon spatially varying local region information. By using local region information, specifically local intensity mean, the RSF model is able to provide desirable segmentation results even in the presence of intensity inhomogeneity. Some related methods were recently proposed in [5, 14, 16, 17, 20, 21], which have similar capability of handling intensity inhomogeneity as the RSF model. These methods are, however, to some extent sensitive to initialization, which limits their practical applications. In a word, in local information based models, the common considerable drawback is the high dependence on initial position of the contour. Besides, in such models, the energy functionals are classically transformed into a level set formulation and then minimized by solving the corresponding Euler-Lagrange equations, which is relatively slow to converge [19].

To improve the robustness to initialization, some hybrid models combining the local and global intensity fitting energies were proposed [15, 16, 24, 25]. The researchers usually combined the local intensity fitting term used in RSF model and an auxiliary global intensity fitting term used in CV model to discover their merits in driving curve evolution in different regions. Specifically, when the contour is far away from object boundaries, the force from the global intensity information is dominant and has large capture range. When the contour is close to the object boundaries, the force from the local intensity information becomes dominant, which attracts the contour toward and finally stops the contour at object boundaries [15]. The weight of the auxiliary global fitting term by using local contrast of the image is dynamically adjusted, which reduced the dependence on the initial contour [16]. The technique of using global image information can improve the robustness to the initialization of contours and performs better than the single fitting term independently. However, in these combined models, when the contour is close to the weak object boundaries, the interference from the global intensity force will result in the deviation of contour from the real object boundary. Therefore, it is necessary to exploit more discriminative energy functionals that incorporate the local and global information from another viewpoint to improve the performance of these combined models.

In this paper, motivated by the region-based models that combine the local and global intensity, we propose a novel region-scalable geometric active contour model for handling the intensity inhomogeneity and weak boundary problems in medical image segmentation. The basic idea of our model is to combine the discriminant information and the fitting information to drive the contour evolution to the desired object. To achieve this, we propose a new region-scalable discriminant and fitting energy functional and extend it to work with geometric active contour in local and global regions. The discriminant term in our model aims at separating background and foreground in scalable regions while the fitting term tends to fit the intensity in these regions. This energy is then incorporated into a variational level set formulation with a level set regularization term for accurate computation. The new model utilizes intensity information in the local and global region as much as possible, so it not only handles better intensity inhomogeneity, but also allows for more robustness to noise and more flexible initialization in comparison to the original region and regional-scalable based models.

Our new model has the following characteristics in comparison to the representative local or global region-based models. First of all, by applying the discriminant information, the defined new energy functional guarantees that the algorithm has the tendency to reach an optimal separation between foreground and background. Second, our new model can control the influence of the local and global region information and discriminant information by adjusting the weights. Finally, our new model is less sensitive to initial contours. We apply this model for synthetic and real medical image segmentation, and experimental results show the advantages of our method in terms of accuracy and robustness.

The reminder of this paper is organized as follows. In Section 2 some well-known region-based models and their limitations are briefly reviewed. In Section 3 the proposed method and its numerical implementation are presented. The experimental results and discussions are presented in Section 4. Some conclusions are given in Section 5.

## 2. Background

### 2.1. The CV Model

Given a gray level image *I* : Ω ⊂ *R* ^2^ → *R*, let *C* be a closed contour in the image domain Ω, which separates Ω into two regions Ω_1_ and Ω_2_. For a given point **x** ∈ Ω, the energy functional in Chan and Vese (CV) model is defined as

(1) $$\begin{array}{c} E^{\text{cv}}\left(C_{1},C_{2},C\right)=\overset{2}{\underset{i=1}{\sum }}\lambda_{i}\int_{\Omega_{i}}{\left(I\left(x\right)-C_{i}\right)}^{2}dx, \end{array}$$

where *C*_*i*_ denotes the mean of the intensity in region Ω_*i*_. *λ*_*i*_ denotes the nonnegative parameters.

CV simplifies the Mumford-Shah functional [26] by assuming that the original image is a piecewise constant function. It has the ability of obtaining a larger convergence range and less sensitivity to the initialization due to the global data fitting energy terms in (1). However, it generally fails to segment images with intensity inhomogeneity. Specifically, the estimation of *C*_*i*_ in CV model is less accurate to represent the complicated distribution in inhomogeneous images. Similarly, more general piecewise constant models are also not good at such images [27].

### 2.2. The RSF Model

To handle intensity inhomogeneity with active contour framework, Li et al. [12, 13] recently proposed a region-scalable fitting (RSF) model in a variational level set formulation. The energy functional in RSF model is defined as

(2) ERSF(f1,f2,C) =∑i=12λi∫Ω∫ΩiK(x−y)(I(y)−fi(x))2dy dx,

where *K*(**x** − **y**) is the window function that has an important localization property [13]. *λ*_*i*_ is positive constant (usually fixed as 1). *f*_*i*_(**x**) is a scalar that approximates the mean of local image intensities in Ω_*i*_. The intensity *I*(**y**) in a local region is centered at the point **x**, whose size is controlled by the nonnegative kernel function *K*. Thus this energy is designed for region-scalable intensity fitting of a contour *C* at a point **x**.

By adjusting the window size, the RSF model can reduce the effect of intensity inhomogeneity in object segmentation. However, this model is sensitive to the initial contour in level set evolution because the approximation of the intensities outside and inside the evolving contour *C* works in a local way. Also, it is easy to fall into local minimum, which limits its practical applications. Additionally, the intensity fitting in local region cannot provide appropriate results in the segmentation tasks of images in the presence of noise and heavy intensity nonuniformity.

## 3. The Proposed Model

In this section, we will detail our region-based active contour model with local and global discriminant and fitting energy for image segmentation. Firstly, we propose the scalable mean discriminant. Then, we extend this discriminant to the local and global versions and integrate them into the CV and RSF level set framework. Finally, we present the implementation details of our model.

### 3.1. Definitions of Region-Scalable Mean Discriminant/Fitting Energy Functional

Let the image region Ω be spitted by the closed contour *C* into two regions: Ω_1_ = inside(*C*) and Ω_2_ = outside(*C*). To obtain the local regions on the two sides of the boundary, a window function *K*(**y** − **x**) is introduced to build a local region centered at **x**. This window function can be flexibly chosen as a truncated uniform function or a Gaussian function. A simple version is as

(3) $$\begin{array}{c} K_{\sigma }\left(y-x\right)=\{\begin{array}{cc} \frac{1}{{\left(2\sigma +1\right)}^{2}}, & \left|x-y\right|\leq \sigma \\ 0, & \left|x-y\right|>\sigma , \end{array} \end{array}$$

where the parameter *σ* denotes the radius of neighborhood close related to the degree of the intensity inhomogeneity. A smaller *σ* is more suitable for an image region with more localized intensity inhomogeneity and vice versa, since the approximation is valid only in a smaller neighborhood. With this window function, we can get the number of pixels in the two distinct regions as *N*_1_^*σ*^(*C*) and *N*_2_^*σ*^(*C*). Also we can estimate the intensity means in both regions as *D*_1_^*σ*^(*C*) and *D*_2_^*σ*^(*C*). Based on these estimations, we propose a region-scalable mean discriminant energy functional as

(4) Jσ(C)=(1N1σ−1∫Ω1(I(x)−D1σ(C))2dx +1N2σ−1∫Ω2(I(x)−D2σ(C))2dx)×((D1σ(C)−D2σ(C))2)−1.

This energy functional implies that the intensity of each local region should be fitted by the estimated mean as much as possible, while the estimated means in different region should be as distinguishable as possible. On the other hand, the upper two terms in this functional can be rewritten as the variance estimation; for example, *V*_1_^*σ*^(*C*) = (1/(*N*_1_^*σ*^ − 1))∫_Ω_1__(*I*(**x**) − *D*_1_^*σ*^(*C*))^2^ *d* **x** and *V*_2_^*σ*^(*C*) = (1/(*N*_2_^*σ*^ − 1))∫_Ω_2__(*I*(**x**) − *D*_2_^*σ*^(*C*))^2^ *d* **x**; in this sense, the quantity *V*_1_^*σ*^ + *V*_2_^*σ*^ is called the within-class scatter of the samples separated by the contour *C*; so (4) presents the inverse formulation of Fisher linear discriminant analysis (LDA). The purpose of LDA is to solve an optimal discriminant vector where examples from the same class are projected very close to each other and simultaneously the projected means are as far as possible. Theoretically, Fisher LDA can minimize Bayes error in the two-class problem under the assumption that all samples obey Gaussian distribution with a common covariance matrix. In this context, the optimal *C* for minimizing *J* ^*σ*^(*C*) can achieve the best classification for distinguishing the object and background.

On the other hand, the RSF model can be rewritten as

(5) Eσ(f1,f2,C) =∑i=12λi∫Ω∫ΩiKσ(x−y)(I(y)−fi(x))2dy dx.

It is seen that *E* ^*σ*^(*f*_1_, *f*_2_, *C*) is identical to the RSF model for small *σ*, while it approaches to the CV model with an additional constant when *σ* → *∞* because *f*_*i*_(**x**) approaches to the mean inside the contour (*i* = 1) and outside the contour (*i* = 2), respectively. Thus we can call this energy as region-scalable mean fitting energy functional.

### 3.2. The Combined Energy Functional

To handle the intensity inhomogeneity problem in image segmentation, we define a new energy functional that simultaneously incorporates the fitting and discriminative information; that is,

(6) E(C)=[(1−w)EG(C)+wEL(C)]+k[(1−w)JG(C)+wJL(C)]+ν|C|,

where *E* ^*G*^(*C*) and *J* ^*G*^(*C*) focus on the global information, while *E* ^*L*^(*C*) and *J* ^*L*^(*C*) focus on the local information. *J* ^*G*^(*C*) aims to extract the global discriminant information by using larger window-size parameter (i.e., *σ*_*G*_), whereas *J* ^*L*^(*C*) works on a local region using small window-size parameter (i.e., *σ*_*L*_). They are linearly combined to extract image fitting information at different scales by the positive parameter *w*  (0 ≤ *w* ≤ 1). When the image intensity is homogeneous, the parameter value *w* should be chosen to be small enough and vice versa. In addition, the parameter *k* (0 ≤ *k* ≤ 1) adjusts the importance of the region-scalable mean discriminant with respect to the region-scalable mean fitting. We will discuss the both parameters in the experimental section. |*C*| denotes the length of contour *C*. As in most of active contour models, the contour *C* is smoothed by penalizing |*C*| with a small nonnegative parameter *ν*. In order to easily handle topological changes, we will convert this energy functional to a level set formulation in the next subsection.

### 3.3. Level Set Formulation

According to the framework of level set methods, the contour *C* is represented by the zero level set of a Lipschitz function *ϕ*(**x**) : Ω → *R*, with the following properties: *ϕ*(**x**) > 0 if **x** ∈ inside(*C*); *ϕ*(**x**) = 0 if **x** ∈ *C*; and *ϕ*(**x**) < 0 if **x** ∈ outside(*C*). Let *H*(*ϕ*(**x**)) be the Heaviside function and separated by *M*_*i*_(**x**); that is, *M*_1_(**x**) = *H*(*ϕ*(**x**)); *M*_2_(**x**) = 1 − *H*(*ϕ*(**x**)); the energy in (4) can be expressed as

(7) Jσ(ϕ)=(1N1σ−1∫Ω(I(x)−D1σ(ϕ))2M1(ϕ(x))dx +1N2σ−1∫Ω(I(x)−D2σ(ϕ))2M2(ϕ(x))dx)×((D1σ(ϕ)−D2σ(ϕ))2)−1.

Similar to the level set formulation of the RSF, the energy in (5) can be expressed as

(8) Eσ(f1,f2,ϕ) =∑i=12λi∫Ω∫ΩKσ(x−y)(I(y)−fi(x))2×Mi(ϕ(y))dy dx.

As in typical level set methods, the penalization on the |*C*| is transformed to regularize the zero level set to derive a smooth contour during evolution; that is,

(9) $$\begin{array}{c} L\left(\phi \right)=\int_{\Omega }\left|\nabla H\left(\phi \right)\right|dx. \end{array}$$

For more accurate computation involving the LSF and its evolution, we need to regularize the LSF by penalizing its deviation from a signed distance function [28], which can be characterized by the following energy functional:

(10) $$\begin{array}{c} R\left(\phi \right)=\frac{1}{2}\int_{\Omega }{\left(\left|\nabla \phi \right|-1\right)}^{2}dx. \end{array}$$

Thus, the entire energy functional in level set formulation is defined as

(11) F(ϕ)=[(1−w)EG(ϕ)+wEL(ϕ)]+k[(1−w)JG(ϕ)+wJL(ϕ)]+νL(ϕ)+μR(ϕ),

where the small positive constant *μ* > 0 stands for the weights of regularization on the LSF *ϕ*.

### 3.4. Energy Minimization

We use the standard gradient descent (or steepest descent) method to minimize the energy functional in (11). Specifically, there are two steps in this minimization process. The first step aims at minimizing the functional *E* ^*σ*^(*f*_1_, *f*_2_, *ϕ*) with respect to *f*_1_ and *f*_2_, for a fixed LSF *ϕ*. In calculus of variations, it is shown that the functions *f*_1_ and *f*_2_ minimizing *E* ^*σ*^(*f*_1_, *f*_2_, *ϕ*) satisfy the Euler-Lagrange equation ∫_Ω_ *K*_*σ*_(**x** − **y**)(*I*(**y**) − *f*_*i*_(**x**))^2^ *M*_*i*_(*ϕ*(**y**))*d* **y** = 0; *i* = 1,2, so we can obtain

(12) $$\begin{array}{c} f_{i}\left(x\right)=\frac{\int_{\Omega }K_{\sigma }\left(x-y\right)I\left(y\right)M_{i}\left(\phi \left(y\right)\right)dy}{\int_{\Omega }K_{\sigma }\left(x-y\right)M_{i}\left(\phi \left(y\right)\right)dy};\;i=1,2. \end{array}$$

This equation can be rewritten as the convolution-like form

(13) $$\begin{array}{c} f_{i}\left(x\right)=\frac{K_{\sigma }\left(x\right)*\left[M_{i}\left(\phi \left(x\right)\right)I\left(x\right)\right]}{K_{\sigma }\left(x\right)*M_{i}\left(\phi \left(x\right)\right)};\;i=1,2. \end{array}$$

This form indicates that *f*_*i*_(**x**)  (*i* = 1,2) is a weighted average of the intensities in a neighborhood of **x**, whose size is proportional to the scale parameter *σ*.

The second step aims at minimizing the functional *J* ^*σ*^(*ϕ*) with respect to *D*_1_^*σ*^(*ϕ*) and *D*_2_^*σ*^(*ϕ*) in condition of fixed LSF *ϕ*. In calculus of variations, it is shown that the functions *f*_1_ and *f*_2_ minimizing *J* ^*σ*^(*ϕ*) also satisfy the Euler-Lagrange equation ∫_Ω_(*I*(**x**) − *D*_*i*_^*σ*^(*ϕ*))^2^ *M*_*i*_(*ϕ*(**x**))*d* **x** = 0; *i* = 1,2, so we obtain

(14) $$\begin{array}{c} D_{i}\left(\phi \right)=\frac{\int_{\Omega }^{}\int_{\Omega }^{}K_{\sigma }\left(x-y\right)I\left(x\right)M_{i}\left(\phi \right)dy\;dx}{\int_{\Omega }^{}\int_{\Omega }^{}K_{\sigma }\left(x-y\right)M_{i}\left(\phi \right)dy\;dx};\;i=1,2. \end{array}$$

To get a specific derivation, we can compute the number of pixels in different regions as *N*_*i*_^*G*^(*ϕ*) = ∫_Ω_ *K*_*G*_(*ϕ*)*M*_*i*_(*ϕ*)*d* **x** and *N*_*i*_^*L*^(*ϕ*) = ∫_Ω_ *K*_*L*_(*ϕ*)*M*_*i*_(*ϕ*)*d* **x**; then we can estimate the region-scalable means of intensities with different parameters in different regions; for example, *C*_*i*_^*G*^(*ϕ*) = ∫_Ω_ *K*_*G*_(*ϕ*)*I*(**x**)*M*_*i*_(*ϕ*)*d* **x**/*N*_*i*_^*G*^(*ϕ*); *C*_*i*_^*L*^(*ϕ*) = ∫_Ω_ *K*_*L*_(*ϕ*)[*I*(**x**)*M*_*i*_(*ϕ*)]*d* **x**/*N*_*i*_^*L*^(*ϕ*); *i* = 1,2. *K*_*G*_ and *K*_*L*_ are the window functions working globally and locally, respectively.

In calculus of variations, this energy functional can be minimized by solving the following gradient flow:

(15) ∂ϕ∂t=−δ(ϕ)[(1−w)(λ1(I−C1G)2−λ2(I−C2G)2)+w(λ3C1L−λ4C2L)]−kδ(ϕ) ×[(1−w)(I−C1G)2/(N1G−1)−(I−C2G)2/(N2G−1)(C1G−C2G)2+w(I−C1L)2/(N1L−1)−(I−C2L)2/(N2L−1)(C1L−C2L)2] +λδ(ϕ)div⁡(∇ϕ|∇ϕ|)+μδ(ϕ)(∇2ϕ−div⁡(∇ϕ|∇ϕ|)),

where *δ*(*ϕ*) is the Dirac function, which is the derivative of the Heaviside function *H*.

### 3.5. Numerical Implementation

In this subsection we briefly present the numerical implementation to solve the evolutions in (15) via a simple, explicit finite difference scheme rather than a complex upwind scheme that is commonly used in conventional level set formulation. Firstly, we employ a flatter function and its derivative approaching to the Heaviside function *H* and the Dirac function *δ*; that is,

(16) $$\begin{array}{c} H_{ɛ}\left(x\right)=\{\begin{array}{cc} \frac{1}{2}\left(1+\frac{2}{\pi }\arctan\left(\frac{x}{ɛ}\right)\right); & \left|x\right|\leq ɛ\\ 1; & x>ɛ\\ 0; & x<-ɛ, \end{array}\\ \delta_{ɛ}\left(x\right)=\frac{1}{\pi }\cdot \frac{ɛ}{{ɛ}^{2}+x^{2}};\;x\in R. \end{array}$$

The *δ*_*ɛ*_(*x*) acts on all level curves, and hence new contours can appear spontaneously, which makes it tend to yield a global minimum [9, 29]. The parameter *ɛ* is usually settled as 1.5.

Secondly, we consider the 2D case with a time-dependent LSF *ϕ*(*x*, *y*, *t*). The spatial derivatives ∂*ϕ*/∂*x* and ∂*ϕ*/∂*y* in our model are approximated by the central difference, and the temporal partial derivative ∂*ϕ*/∂*t* is discretized as the forward difference. Let Δ*t* be the time step, Δ*h* the space step, (*x*_*i*_, *y*_*i*_) = (*i*Δ*h*, *j*Δ*h*) the grid points, and *ϕ*_*i*,*j*_^*n*^ = *ϕ*(*x*_*i*_, *y*_*i*_, *n*Δ*t*) an approximation of *ϕ*(*x*, *y*, *t*) with *n* ≥ 0,  *ϕ* ^0^ = *ϕ*_0_. Set the iteration number *k* > 0 and start with initial LSF *ϕ*_*i*,*j*_^0^; the evolving process can be approximated by

(17) $$\begin{array}{c} \phi_{i,j}^{k+1}=\phi_{i,j}^{k}+\Delta tL\left(\phi_{i,j}^{k}\right), \end{array}$$

where *L*(*ϕ*_*i*,*j*_^*k*^) corresponds to numerical implementation of the right-hand side of (15). Specifically, in the *k*th iteration, the first-order and second-order central differences at *ϕ*_*i*,*j*_ are expressed as *ϕ*_*x*_ = (*ϕ*_*i*+1,*j*_^*k*^ − *ϕ*_*i*−1,*j*_^*k*^)/2Δ*h*; *ϕ*_*y*_ = (*ϕ*_*i*,*j*+1_^*k*^ − *ϕ*_*i*,*j*−1_^*k*^)/2Δ*h*; *ϕ*_*xx*_ = (*ϕ*_*i*+1,*j*_^*k*^ − 2*ϕ*_*i*,*j*_^*k*^ + *ϕ*_*i*−1,*j*_^*k*^)/(Δ*h*)^2^; *ϕ*_*yy*_ = (*ϕ*_*i*,*j*+1_^*k*^ − 2*ϕ*_*i*,*j*_^*k*^ + *ϕ*_*i*,*j*−1_^*k*^)/(Δ*h*)^2^; and *ϕ*_*xy*_ = (*ϕ*_*i*+1,*j*+1_^*k*^ + *ϕ*_*i*−1,*j*−1_^*k*^ − *ϕ*_*i*−1,*j*+1_^*k*^ + *ϕ*_*i*+1,*j*−1_^*k*^)/(Δ*h*)^2^. In this efficient way, we can obtain ∇^2^ *ϕ* = *ϕ*_*xx*_ + *ϕ*_*yy*_ and div⁡(∇*ϕ*/|∇*ϕ*|) = (*ϕ*_*xx*_ *ϕ*_*y*_^2^ − 2*ϕ*_*xx*_ *ϕ*_*x*_ *ϕ*_*x*_ + *ϕ*_*yy*_ *ϕ*_*x*_^2^)/(*ϕ*_*x*_^2^ + *ϕ*_*y*_^2^)^3/2^ to represent the corresponding terms in (15). It is noted that given spacial step Δ*h* = 1,  Δ*t* for this finite difference scheme must satisfy the Courant-Friedrichs-Lewy (CFL) condition *μ*Δ*t* < 0.25. Similar to the reason pointed out by Li et al. [28, 30], the added level set regularization term and the corresponding numerical scheme in our model are stable without the need for reinitialization.

Finally, the main steps of our algorithm are summarized as follows.

**Step 1 .**

Initialize the level set function *ϕ* to a binary function *ϕ*_0_.

**Step 2 .**

Update the LSF using (17).

**Step 3 .**

Check whether the evolution has converged. If either of the zero crossing points stop varying for consecutive iterations or exceed a prescribed maximum number of iterations, stop the iteration; otherwise, go to Step 2.

## 4. Experimental Results

In this section we report the performance of the proposed model with various synthetic and real images from different modalities. We compared our method with relative methods such as Chan-Vese (CV) [9], region-scalable fitting (RSF) [13], and local and global intensity fitting (LGIF) [15]. These models mainly incorporate the global, local, locally. and globally combined intensity information. We tested the proposed method with the following parameters: Δ*t* = 0.1; *λ*_1_ = *λ*_2_ = 1. To keep the consistency between information of image and the criterion, we make the weight of the terms on the local or global information identical. The kernel size in Gaussian window or binary window is *σ* = 3 for local region, and *σ* is settled enough to cover the whole image as a global region. In this case, the CV model can be incorporated as the global term. To speed up the contour evolution, the initial contours are set to a binary function whose values are ±2 inside and outside the contour, respectively. The detailed values of parameters for the different experimental images will be given in the following subsections. All experiments are performed on a Notebook with Pentium Dual-Core CPU 2.3 GHz and 2.0 G RAM, using Matlab 2010b.

### 4.1. Experiments on Synthetic Images

**Figure 1:**
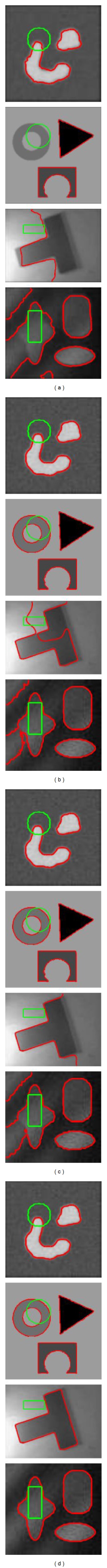
Segmentation results on images without and with intensity inhomogeneity of CV (a), RSF (b), LGIF (c), and our method (d) with different initial contours. The initial contours and the final contours are plotted as green contours and red contours, respectively (color online).

We firstly evaluate our method in segmenting a publicly available synthetic image with intensity inhomogeneity and compare it with CV, RSF, and LGIF methods. The searching ranges of the parameters are as follows: *w* = 0.95 ~ 0.99; *k* = 0.1~0.3; *ν* = 0.001∗255^2^ ~ 0.005∗255^2^; Δ*t* = 0.1; and *μ* = 0.1 ~ 0.2/Δ*t*. The original images marked with the segmentation results with different initial contours are reported in Figure 1. From where it is seen when there is a clear object in the slight obscure background, all the methods can capture right object (e.g., images in the first row). When the objects and background are, respectively, homogeneous but the intensities vary among the objects, the local and combined methods can localize the objects but the global method failed (e.g., images in the second row). Furthermore, when there are objects corrupted by the strong nonuniform noise, only our proposed method extracts the desirable objects. This experiment validates the merits of locally and globally combined method. Due to the global minimization, the segmentation results in the first column by CV model are very similar with respect to different initial contours. However, only the ellipse-like object is correctly segmented. In contrast, the RSF model can successfully segment the ellipse-like and rectangle-like objects due to its capability in capturing the local intensity. However, it fails to extract the star-like object that is full much stronger nonuniform noise than others. In addition, the RSF model is sensitive to the initial contours. The LGIF model outperforms RSF model in its robustness on different initial contours. Unfortunately, it still fails to segment the star-like object. As a comparison, our method successfully extracts three objects under different initial contours; meanwhile, it obtains very similar final contours in these testings due to its global optimization.

**Figure 2:**
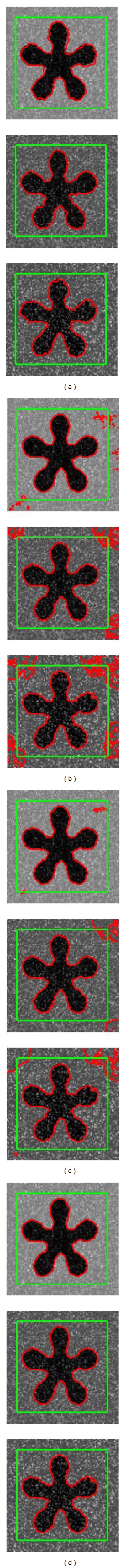
Segmentation results on an image with different noise of CV (a), RSF (b), LGIF (c), and our method (d). The initial and the final zero level sets are plotted as the green and the red contours, respectively. The images in each row are with Gaussian noise characterized by zero-mean and variance 0.01, 0.05, and 0.1, respectively (color online).

We further evaluate the ability of our method in segmenting image with different noise levels. As seen in Figure 2, the testing images are built from a publicly available synthetic image with different zero-mean and variance (0.01, 0.05, and 0.1) Gaussian noise. The parameters are settled as *w* = 0.98; *k* = 0.3; *ν* = 0.002∗255^2^; Δ*t* = 0.1; and *μ* = 0.2/Δ*t*. The kernel size in Gaussian window or binary window is *σ* = 4 for local region, and *σ* is settled enough to cover the whole image as a global region, similar to the above experiments. The length regularization term is fixed for all methods. The results of the compared methods are reported in Figure 2. From where it is seen when the image is less noisy, each method can obtain exact object, and some noise in background are captured as objects for RSF and LGIF. Along with the heavy noise, the CV model can extract object from noisy background but the contour seems less accurate, while the RSF and the LGIF models still produce missegmentation due to their local fitting property. In contrast, our method consistently captures the right boundary of the object. Although our method also employs the local fitting energy in a similar way, it additionally incorporates the region-scalable mean discriminant energy functional that enlarges the difference of object and background; so it produces the desirable segmenting contours in this case.

### 4.2. Experiments on Medical Images

**Figure 3:**
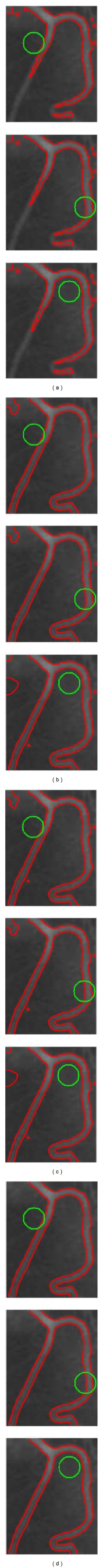
Segmentation results on an X-ray vessel image by CV (a), RSF (b), LGIF (c), and our method (d) with different initial contours. The initial and the final zero level sets are plotted as the green and the red contours, respectively (color online).

**Figure 4:**
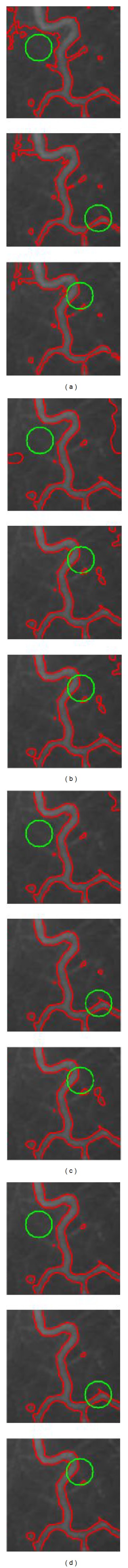
Segmentation results on an X-ray vessel image with heavy noise by compared methods: CV (a), RSF (b), LGIF (c), and our method (d) with different initial contours. The initial and the final zero level sets are plotted as the green and the red contours, respectively (color online).

In this subsection, we employ our method to segment typical medical images with different modalities and compare it to related methods.  Figure 3 shows the results of an X-ray image of blood vessels. The parameters are settled as *w* = 0.95~0.99; *k* = 0.1~0.3; *ν* = 0.001∗255^2^ ~ 0.005∗255^2^; Δ*t* = 0.1; and *μ* = 0.2/Δ*t*. As illustrated in these figures, with different initial contours, the CV model fails to segment the objects, especially in the weak boundary of the vessel. The RSF model is not only sensitive to the initial contours, but also less capable of excluding the false objects. As shown in the figures in the first and second columns, even when the initial contour is settled on the right vessel object, it still captures the wrong contours in the background as pseudo object. The LGIF method performs similar to the RSF method because the local intensity fitting-based energy functional plays more important role than the CV model in segmenting this image. The vessel and additional small background noise are extracted as objects. Additional postprocessing should be used for removing them. This fact limits its availability in practice. In contrast, our method is less sensitive to the initial contour and it captures the vessel accurately in all the cases. The robustness of our method is furtherly verified in the experiment on segmenting a vessel image with heavy intensity inhomogeneity. As shown in Figure 4, the segmentation results of the RSF model are different along with the different initial contours. Remarkable missegmentation appears in the three cases. The LGIF model extracts the vessel in all these cases, but with the false vessel segmented from background, which means that the simple combination of local and global intensity fitting is hard to segment the object in noisy background. In contrast, the proposed model not only gives accurate results but also shows robustness to different initial contours. For example, in the third row of Figure 3 and the first row of Figure 4, even though the initial contours do not contain any foreground objects, our method can still obtain precise segmentation results. These two experiments illustrate the advantages of our proposed model in handling real image: the ability to handle weak boundaries and complex background and robustness to noise.

**Figure 5:**
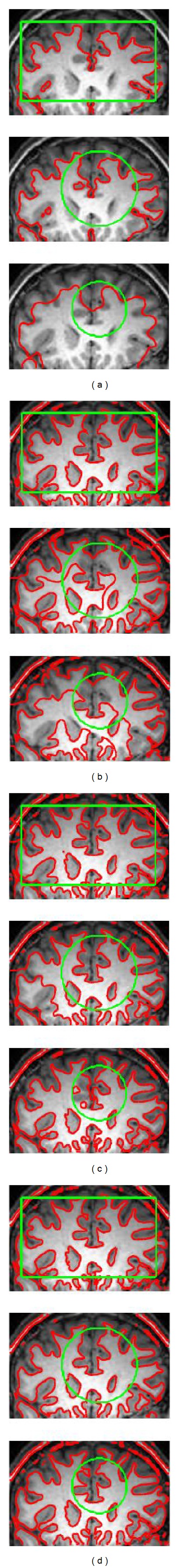
Segmentation results on an MR brain image with intensity inhomogeneity by CV (a), RSF (b), LGIF (c) and our method (d) with different initial contours. The initial and the final zero level sets are plotted as the green and the red contours, respectively (color online).

**Figure 6:**
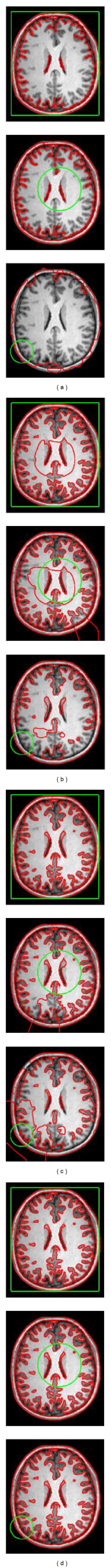
Segmentation results on an MR brain image with intensity inhomogeneity by CV (a), RSF (b), LGIF (c), and our method (d) with different initial contours. The initial and the final zero level sets are plotted as the green and the red contours, respectively (color online).

We further evaluate our method in segmenting MRI brain images with intensity inhomogeneity. These images usually not only contain weak boundaries between gray matter and white matter due to low contrast and partial volume effect, but also present intensity inhomogeneity arisen from the acquisition style. The parameters are searched in the range *w* = 0.1~0.99; *k* = 0.3; *ν* = 0.001∗255^2^ ~ 0.005∗255^2^; Δ*t* = 0.1; and *μ* = 0.2/Δ*t* for an optimal segmentation of each method. Figures 5 and 6 present the performance of our method and compared methods on segmenting two MR brain images with remarkable intensity inhomogeneity. The intensities of white matter in Figure 5 have visible intensity variations and are obscure in most cortex regions. In this experiment, Both CV and RSF models cannot segment the details of the white matter accurately. The results of LGIF are slightly better than those of CV and RSF models, but there are still some segmentation errors, especially in different initial contours. Our method outperforms them in successfully segmenting the white matter in the region with nonuniform intensities. The white matter object in Figure 6 is not corrupted by the intensity inhomogeneity but is more complex in the cortex region. In this experiment, it is observed that our model yields more accurate results than other methods. The experimental results again illustrate the merit of our proposed method: the abilities to deal with intensity inhomogeneity, weak boundaries and complex background, and robustness to noise. These MR images are rather noisy and the object boundary is very weak. As a combined model, LGIF performs better than CV and RSF models; however, it is still easy to misclassify the gray matter as white matter. Again, this fact verifies the limitation of simple combination of local information and global information to drive the curve evolution. On the contrary, the mean discriminant energy functional in our model plays more important role in driving the curve evolution rightly.

### 4.3. Quantitative Experiments on Medical Images

**Figure 7:**
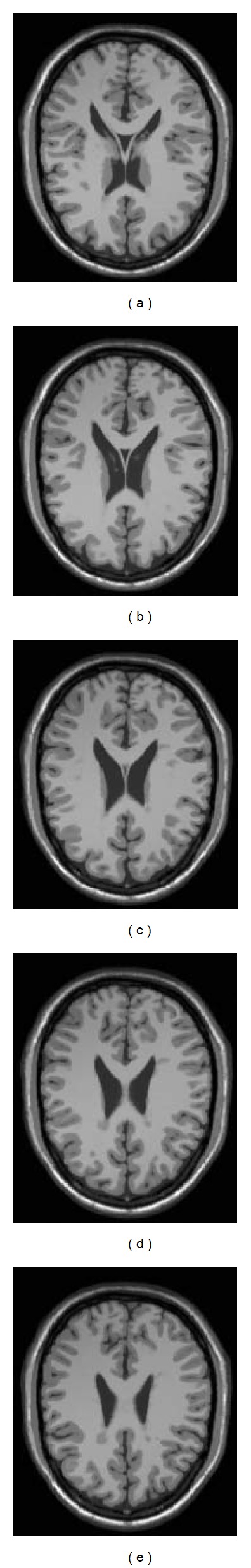
Testing images in quantitative experiments.

To quantitatively evaluate the performance of our proposed model, we use it to segment five brain MRI images of ground-truth object (i.e., white matter) and compare it to CV, RSF, and LGIF methods. The 2D MR images are taken from McGill Brain Web [31] with 3% noise level and 40% intensity nonuniformity. The space resolution in *x* and *y* directions is 1 mm. As seen in Figure 7, these images are characterized as low contrast, intensity inhomogeneity, weak object boundary. Some preprocessing such as filtering and intensity adjustment skull stripping were taken. We employ the Jaccard similarity index (JS) to quantitatively measure the segmentation performance of these methods. This JS index is the ratio between intersectional area of *S*_1_ and *S*_2_ and their united area; that is,

(18) $$\begin{array}{c} J\left(S_{1},S_{2}\right)=\frac{\text{area}\left(S_{1}\cap S_{2}\right)}{\text{area}\left(S_{1}\cup S_{2}\right)}. \end{array}$$

The closer the *J* is to 1, the more similar *S*_1_ is to *S*_2_. In our experiments, *S*_1_ is the segmented region by the four compared methods, and *S*_2_ is the ground truth. For each method, we used its optimal initial contour to obtain their best performance. Other common parameters in these methods were settled as same as possible for fair comparison, except the iteration number and the exclusive parameters of each method. The evolution iteration will stop once the difference of two neighboring contours of the zero level set is less than ten pixels. It should be noted that the larger Δ*t* can speed up the evolution of level sets in these methods but easily makes boundary leakage or zero LS disappear; so we put the segment accuracy as the first place during the selection of these parameters, for example, Δ*t* = 0.05~0.2. Other parameters are searched in *ν* = 0.001∗255^2^ ~ 0.003∗255^2^ with step 0.001∗255^2^; *μ* = 0.2/Δ*t*; *w* = 0.8~0.99 with step 0.05; *k* = 0.01~10 with step 0.05. To obtain fair experimental results, the programs were conducted 50 times under same condition; then the average of the JS values and CPU time consuming is computed as indices for measuring the efficiency of each method. The compared methods were programmed just based on the original papers and not algorithmic optimization or programming technique was taken into consideration; neither narrow band constriction nor other skills were incorporated into the compared methods.

**Figure 8:**
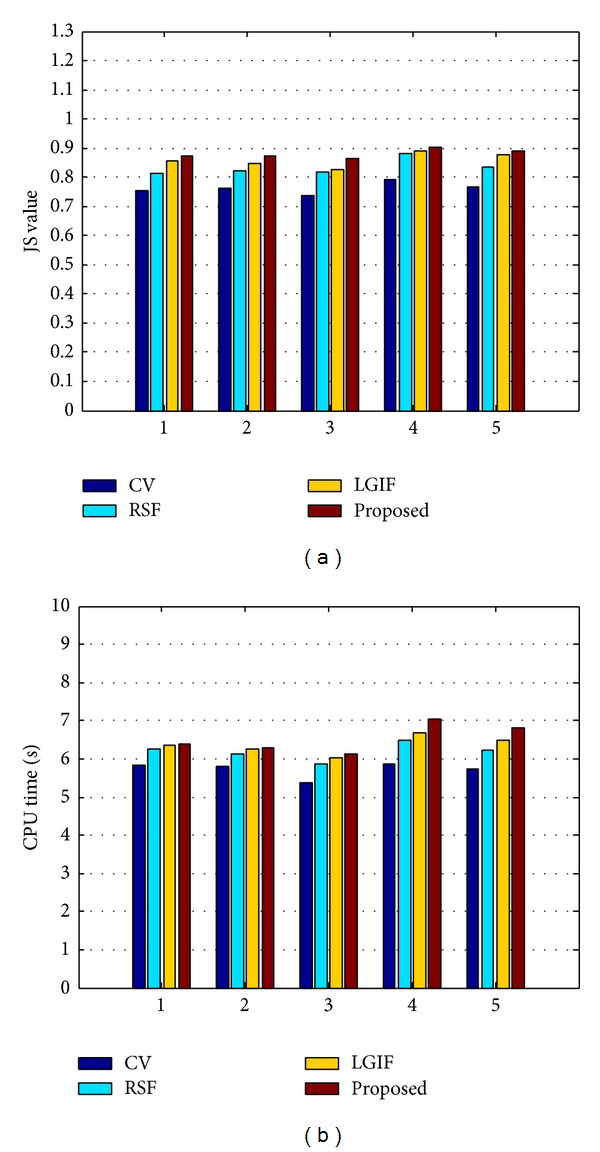
JS values (a) and CPU times (b) of CV, RSF, LGIF, and the proposed methods. The *x*-axis denotes the index of image.

The JS indices and the average CPU time consumption (in seconds) by applying the four compared methods are reported in Figure 8, from where it is seen the JS value of our model is larger than those of other three methods. It indicates that the segmentation precision of our model is superior to the other three models. Furthermore, the following can be found.

1. The JS values of our model and LGIF model are remarkably higher than those of RSF model and CV model. This means that the models combining local and global information (e.g., our method and LGIF model) can achieve better segmenting accuracy than the methods singly using local or global information (e.g., RSF model and CV model).
2. The JS value of our model is higher than LGIF model and that of LGIF model is higher than those of RSF model and CV model. Which indicates that although the combined model by incorporating the local intensity fitting and global intensity fitting can improve the segmentation accuracy, the energy functional that incorporates the local and global mean discriminant information is also desirable to drive the curve evolution. In this case the local information is dominant while the global information plays an auxiliary role due to their large weights and small weights.
3. The JS value of CV model is remarkably lower than those of RSF, LGIF, and our proposed models using local information. It means, in the task of segmenting images with intensity inhomogeneity, that the local information plays much more important role than global information. So in our future work, it is still necessary to emphasize the local information during investigating the energy functional for the segmentation of images full of intensity inhomogeneity.
4. As reported in the right subfigure of Figure 8, the computation efficiency of our method is very close to those in RSF and LGIF models and slightly more than that of CV model. This is mainly due to the computation of local information being more time consuming than the global information.

## 5. Conclusions

In this paper, we have presented a region-based active contour model with region-scalable mean discriminant and fitting energy for image segmentation. The core contribution of this work lies in that we introduce the region-scalable discriminant and fitting energy functionals and extend them to work in local and global regions. We also transform them into a variational level set framework using calculus of variations technique, which make its availability for other level set-based applications. Comparative segmentation experiments on image with intensity inhomogeneity show that our method can achieve accurate segmentation results with various initial contours. Quantitative comparison also demonstrates that our model is competent in terms of accuracy.

It should be noted that although introducing region-scalable mean discriminant can improve the robustness of our model, the initialization may still slightly affect the final solution of our model due to the nonconvexity of the energy functional. So it is more desirable to design a convex energy to drive the evolution of level set function directly. In addition, for the image with strong bias field, our model needs further improvements in the weak boundary, especially in the weight adjustment between local and global regions. They will be the future work in our study.
